# Effects of Tocotrienol on Cardiovascular Risk Markers in Patients With Chronic Kidney Disease: A Randomized Controlled Trial

**DOI:** 10.1155/jnme/8482883

**Published:** 2025-01-13

**Authors:** Liana Trugilho, Lívia Alvarenga, Ludmila Cardozo, Bruna Paiva, Jessyca Brito, Isis Barboza, Jonatas Almeida, Juliana dos Anjos, Pramod Khosla, Marcelo Ribeiro-Alves, Denise Mafra

**Affiliations:** ^1^Graduate Program in Medical Sciences, Fluminense Federal University (UFF), Niterói, Rio de Janeiro, Brazil; ^2^Physiology-Graduate Program in Biological Sciences, Federal University of Rio de Janeiro (UFRJ), Rio de Janeiro, Brazil; ^3^Graduate Program in Nutrition Sciences, Fluminense Federal University (UFF), Niterói, Rio de Janeiro, Brazil; ^4^Graduate Program in Cardiovascular Sciences, Fluminense Federal University (UFF), Niterói, Rio de Janeiro, Brazil; ^5^Clinic Unit of Research, Fluminense Federal University (UFF), Niterói, Rio de Janeiro, Brazil; ^6^Department of Nutrition and Food Science, Wayne State University, Detroit, Michigan, USA; ^7^HIV/AIDS Clinical Research Center, National Institute of Infectology Evandro Chagas (INI/Fiocruz), Manguinhos, Rio de Janeiro, Brazil

**Keywords:** chronic kidney disease, dyslipidemia, inflammation, NF-*κ*B, NRF2, oxidative stress, tocotrienol

## Abstract

Tocotrienols, isomers of vitamin E, may provide an effective nutritional strategy to mitigate common cardiovascular risks such as dyslipidemia, inflammation, and oxidative stress in patients with chronic kidney disease (CKD). This double-blind, placebo-controlled, randomized clinical trial aimed to evaluate the effects of a tocotrienol-rich fraction (TRF) supplementation (300 mg/day) on oxidative stress and inflammatory markers, including transcription factors in nondialysis (ND) and hemodialysis (HD) CKD patients for three months. Interleukin-6, tumor necrosis factor-*α* (IL-6 and TNF-*α*), C-reactive protein (CRP), lipid peroxidation, biochemical parameters, and transcription factors such as NRF2 and NF-*κ*B mRNA expression were evaluated. Seventeen HD patients (9 in the placebo group, 8 in the TRF group) and 16 ND CKD patients (8 in the placebo group and 8 in the TRF group) completed the study. In HD patients, significant reductions were observed in LDL cholesterol (*p*=0.04) and total plasma cholesterol levels (*p*=0.01) after TRF intervention. CRP serum levels decreased significantly in ND CKD patients (*p*=0.05) after TRF supplementation. Transcription factors NRF2 and NF-*κ*B mRNA expressions remained unaltered in both groups. This study suggests that TRF supplementation may mitigate dyslipidemia and inflammation, factors involved with increased cardiovascular risk, in CKD patients, with variations in efficacy between HD and ND patients.

**Trial Registration:** ClinicalTrials.gov identifier: NCT04900532

**Summary**

• TRF supplementation may mitigate oxidative stress and inflammatory markers in patients with CKD.
• Hemodialysis patients experienced significant reductions in LDL cholesterol and total plasma cholesterol levels after 3 months of TRF supplementation.
• Nondialysis patients showed significantly decreased C-reactive protein serum levels after TRF supplementation.
• TRF supplementation may be a valuable nutritional strategy for mitigating dyslipidemia and inflammation, factors associated with increased cardiovascular risk in CKD patients.

## 1. Introduction

Chronic kidney disease (CKD) is a worldwide public health concern [1]. Its progression to end stages culminates in kidney substitutive therapy, which leads to a substantial reduction of life quality and elevates the risk of cardiovascular outcomes, a significant cause of mortality among CKD patients [2]. The pathogenesis and progression of CKD involve complex inflammatory processes related to oxidative stress [3]. Individuals with CKD present elevated levels of key inflammatory and oxidative markers, including interleukin-6 (IL-6), tumor necrosis factor-*α* (TNF-*α*), and C-reactive protein (CRP), and malondialdehyde (MDA), which may increase with the glomerular filtration decline and increased cardiovascular disease risk [4].

Individuals with CKD present an imbalance between reactive oxygen species and antioxidant defenses that results in the upregulation of nuclear factor kappa B (NF-*κ*B) gene expression, leading to the recruitment of immune cells and the expression of pro-inflammatory genes, thereby contributing to inflammation [5]. Conversely, these individuals present downregulation of the expression of erythroid nuclear factor 2 related to factor 2 (NRF2) mRNA, responsible for the activation of cytoprotective genes and antioxidant enzymes, including glutathione-S-transferases, superoxide dismutase, catalase, NAD (P) H quinone oxidoreductase-1, as well as stress response proteins such as heme oxygenase-1 and oxygenase-2, metallothioneins, and heat shock proteins, therefore representing an essential role in defense against inflammation and oxidative stress [6, 7].

Additionally, contributing to cardiovascular risk, patients with CKD exhibit dyslipidemia, characterized by hypertriglyceridemia, impaired high-density lipoprotein (HDL) cholesterol, and variable levels of low-density lipoprotein (LDL) cholesterol, which enhance susceptibility to oxidation [8]. The distinct dyslipidemia profile of this population contributes to atherogenic complications and endothelial dysfunction that promote cardiovascular complications and kidney damage [9].

Managing dyslipidemia, inflammation, and oxidative stress in CKD patients is crucial for mitigating kidney disease progression and reducing cardiovascular morbidity and mortality. Therefore, supplementation with vitamin E, especially tocotrienol, is a potential adjunctive therapy due to its antioxidant and anti-inflammatory properties. It helps modulate lipid metabolism and improve endothelial function in renal blood vessels by reducing oxidative stress and inflammation [10, 11].

Tocotrienol compounds comprise four homologs from the vitamin E family, with three double bonds between isoprenoids in its chain. They may be more easily incorporated into cell membranes compared to tocopherols [12, 13]. They also present a nonenzymatic activity and donate phenolic hydrogen to the peroxyl radical, interrupting the oxidation cascade and contributing to the antioxidant defense [14]. Additionally, tocotrienols can improve lipid metabolism by modulating its involved enzymes and decreasing fatty acid synthase in patients with dyslipidemia [15].

Scientific evidence supporting vitamin E supplementation in CKD patients remains controversial and inconclusive [16]. Vitamin E, particularly tocotrienol-rich fraction (TRF), has gained attention due to its potent antioxidant and anti-inflammatory properties. Unlike tocopherols, tocotrienols are more readily incorporated into cell membranes and exhibit distinct biochemical properties, including their ability to interrupt lipid peroxidation and modulate enzymes related to lipid metabolism. This study builds on prior findings that indicate the potential of TRF to improve lipid profiles, reduce inflammation, and enhance endothelial function [17]. Despite the well-known inflammation and oxidative stress as targets for slowing disease progression, current methods for addressing them in CKD remain limited. Given the shortcomings of existing treatments and the insufficient research into tocotrienols' role in CKD, this study aims to fill critical gaps by evaluating the effects of TRF supplementation on cardiovascular risk markers in patients with CKD who are undergoing hemodialysis (HD) or have early-stage disease. This investigation lays the groundwork for using TRF as a complementary strategy to manage cardiovascular risks in this population.

## 2. Methods

### 2.1. Patients and Ethical Aspects

This double-blind, placebo-controlled, randomized clinical trial was conducted with patients from the Renal Nutrition Outpatient Clinic of the Faculty of Nutrition at Fluminense Federal University (UFF), Niterói, Rio de Janeiro, and the Davita clinic, Itaboraí, Rio de Janeiro, Brazil. The study was carried out after approval by the Ethics Committee of the Faculty of Medicine/UFF (3.105.517).

### 2.2. Inclusion and Exclusion Criteria

Inclusion criteria were age between 18 and 65 years old, estimated glomerular filtration rate (eGFR) between 15 and 60 mL/min for ND patients, and more than 6 months undergoing HD in renal replacement therapy patients. Patients with common comorbidities in CKD, such as diabetes mellitus and hypertension, were eligible. Noninclusion criteria were pregnancy, smoking habits, antibiotics used in the prior 3 months, use of antioxidant supplements, vitamin C, multivitamins, and diseases such as autoimmune, infectious diseases, cancer, liver diseases, or AIDS.

### 2.3. Study Design

After computerized randomization in a 1:1 ratio, performed by an independent statistician using the RStudio Version 1.1.463 program (RStudio, PBC, Boston, MA, USA), participants were allocated into different groups to receive capsules containing TRF or placebo. To maintain the study's double-blindness, an external researcher designated capsules as pumpkin or pepper until the end of the study.

The capsules containing TRF derived from red palm oil were provided by Carotino Sdn (Bhd., Johor, Malaysia). The placebo soft gels were formulated by Kamata Co. Ltd (Tokyo, Japan). The experimental capsules contained 150 mg of TRF with *α*-, *β*-, *δ*-, and *γ*-tocotrienols constituting 27.5%, 3.6%, 9.4%, and 34.3% of total tocotrienols, respectively. They also contained 25% *α*-tocopherol. The placebo soft gels comprised 0.22 mg of tocotrienols in wheat-germ oil. Capsules were consumed twice daily after the main meals (lunch and dinner), totaling 300 mg per day of TRF for 3 months. We sought to revisit the methodology employed by Daud et al. [18], adjusting the TRF dose to evaluate its efficacy on inflammatory and oxidative stress markers. The adherence to TRF supplementation was constantly reminded in each visit and via telephone calls and text messages. At the end of the study, the remaining capsules were counted. The difference between the capsules offered at the beginning of the research and the capsules left over at the end was used to establish each patient's adherence and to exclude those whose consumption was lower than 80%.

### 2.4. Blood Sample Collection

Blood was collected from ND patients by a qualified professional at the Faculty of Nutrition at UFF. The nursing team collected blood from HD patients immediately after the fistula puncture. Samples were collected after a 12-h fast in Vacutainer (Becton, Dickinson and Company, Franklin Lakes, NJ, USA) tubes containing ethylenediaminetetraacetic acid with anticoagulant (1.0 mg/mL) to obtain plasma or clot activator and separator gel to obtain serum and were immediately refrigerated and transported to the clinical research unit of Antônio Pedro University Hospital (Niterói, Rio de Janeiro, Brazil) for analysis. The tubes were centrifuged (15 min, 3000x g, 4°C), and the samples were stored in polypropylene microtubes at −80°C until analysis.

### 2.5. Routine Biochemical Parameters, Nutritional Status, and Dietary Assessment

Routine biochemical parameters such as urea, creatinine, glucose, triglycerides, total cholesterol, HDL albumin, parathyroid hormone (PTH), hemoglobin, ferritin, serum iron, calcium, phosphorus, potassium, and CRP were measured by an automatic biochemical analyzer (Bioclin, Belo Horizonte, Brazil). LDL was calculated using the Friedewald formula [19].

The nutritional status was evaluated using the body mass index (BMI), calculated as body weight divided by the square of the height. The classification of BMI was established according to the World Health Organization [20]. Dietary intake was evaluated through a 24-h dietary recall. Energy and nutrient values were calculated using Brazilian food composition tables [21, 22].

### 2.6. Inflammatory Cytokines Analysis

For the analysis of the inflammatory cytokines IL-6 and TNF-*α*, commercial ELISA kits were used. Following a standard protocol, the 31–2000 pg/mL range was used to measure TNF-*α* and IL-6 concentrations using specific quantitative sandwich ELISA kits (PeproTech Inc., Rocky Hill, NJ, USA). Human TNF-*α* and IL-6 capture antibodies were diluted in PBS at 1/100 (approximately 1 μg/mL) to coat a microplate (Maxisorp-Nunc, Fisher Scientific, Leicestershire, England) with 96 wells overnight. The plates were washed with PBS +0.05% Tween (Sigma-Aldrich, St. Louis, MO, USA) and blocked with PBS +1% BSA and standard samples for 2 h at room temperature. Then, 100 µs of the samples were added. A dilution of 1 per 660 was conducted, and detection antibodies were incubated in the plates for 2 h. Later, the streptavidin-HRP conjugate was diluted (1/2000) and incubated (100 μL per well) at room temperature for 30 min. CoA tetramethylbenzidine (Sigma-Aldrich, St. Louis, MO, USA) was used as a substrate for 20 min to confer color to the reaction. 1 M HCl was used to stop the reaction. An automatic plate reader read the optical density at 450 nm, with correction at 650 nm. A standard curve was constructed, and samples were quantified if the absorbance was in the linear portion of the standard curve.

### 2.7. Oxidative Stress Marker Analysis

The reaction between MDA and thiobarbituric acid was used as a marker of lipid peroxidation using the modified Ohkawa method [23]. The samples were diluted in microtubes with thiobarbituric acid (0.6% *w*/*v*), SDS (8.1% *w*/*v*), and phosphoric acid (1% *w*/*v*), then heated to 95°C for 60 min. The microtubules were centrifuged at 4000 rpm for 20 min at 20°C, and the supernatant was separated. The absorbance was measured using a Synergy H1M microplate reader (BioTek, Winooski, VT, USA) at 532 nm.

### 2.8. Real-Time PCR

The whole blood sample was transferred to a Falcon tube with saline solution (Hanks' Balanced Salt solution, Sigma-Aldrich, St. Louis, MO, USA) in a 1:1 ratio. After being homogenized, samples were carefully transferred to another tube containing histopaque (Sigma-Aldrich). The samples were centrifuged at 1700 rpm for 30 min at 18°C to obtain the cell suspension concentrate, which was transferred to a new Falcon tube and added saline solution. The samples were centrifuged again at 1700 rpm for 5 min at 18°C, and the resulting supernatant was discarded. The formed pellet was resuspended in saline solution and centrifuged for 10 min at 2000 rpm at 18°C to obtain the nuclear content. The total RNA with the Total SV RNA Isolation System (Promega, Madison, WI, USA) could be extracted. A subsequent cDNA was synthesized using the High-Capacity cDNA Reverse Transcription kit (Thermo Fisher Scientific, Waltham, MA, USA). TaqMan Gene Expression (Thermo Fisher) assays for the detection of NRF2 (Hs00975961_g1), NF-*κ*B (Hs00765730_mL), and GAPDH (Hs02758991_g1) control mRNA expression was used. The Prism 7500 Sequence Detection System ABI (Applied Biosystems, Foster City, CA, USA) and the standard cyclic conditions were used for PCR amplification. NRf2 and NF-*κ*B mRNA expression was normalized against GAPDH, and the expression level was calculated using the ΔΔCT (double delta threshold cycle) method.

## 3. Outcomes

The primary outcome was mRNA expression of transcription factors NRF2 and NF-*κ*B, particularly an increase in NRF2 and a decrease in NF-*κ*B from baseline to month 3, to evaluate inflammatory and oxidative response to these transcription factors. Secondary outcomes included improvement in IL-6, TNF-*α*, and CRP as inflammatory response indicators and improvement in MDA as a marker of oxidative stress. Additional analyses were conducted to assess improvements in biochemical parameters and renal function.

### 3.1. Statistical Analysis

Analyses were conducted using R software v.4.2 (R Foundation for Statistical Computing, Vienna, Austria), employing packages such as “lme4”, “emmeans”, and their dependencies. Continuous numerical baseline demographic and clinical variables were compared using nonparametric Mann–Whitney *U* tests, while Chi-squared tests were applied to categorical variables. The expression of mRNA for transcription factors and plasma levels of cytokines and oxidative stress markers were considered outcome variables and log-transformed when necessary.

The primary outcomes were analyzed using linear mixed-effects models to assess time-intervention interactions, considering participants as random effects. Secondary outcomes were evaluated using multiple linear fixed-effects models to compare changes from baseline to the 3-month endpoint. These models were adjusted for potential confounding variables, such as age, sex, and BMI. Expected mean marginal values were estimated by holding covariates at their mean or representative values, and contrasts were constructed. Pairwise comparisons were corrected using the Tukey honest significant difference method, and *p* values < 0.05 were deemed statistically significant.

Effect sizes were calculated for key findings to interpret the results better. For continuous variables, Cohen's *d* was computed as the mean difference divided by the pooled standard deviation, and 95% confidence intervals (CIs) were calculated to provide context for the precision of these estimates.

## 4. Results

**Figure 1:**
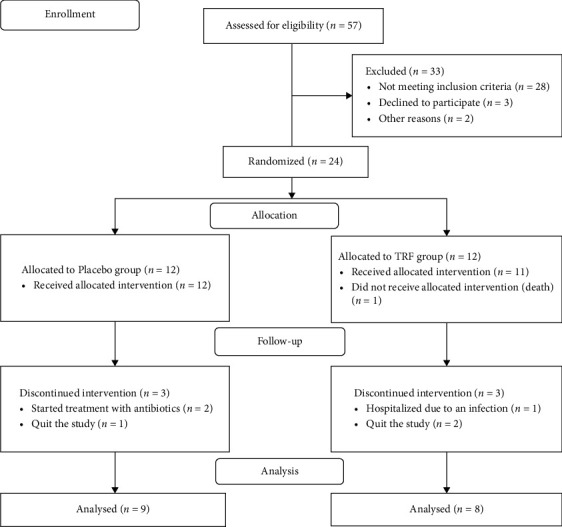
Flow diagram of the study regarding TRF supplementation in patients with chronic kidney disease undergoing hemodialysis (HD).

**Figure 2:**
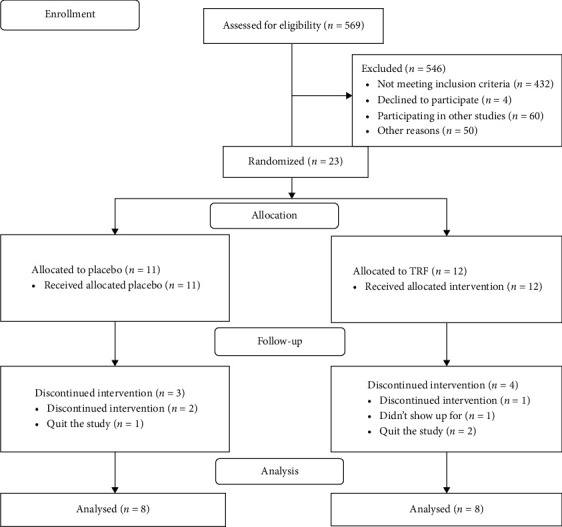
Flow diagram of the study regarding TRF supplementation in non–dialysis (ND) patients with chronic kidney disease.

Seventeen HD patients (9 in the placebo group, 8 in the TRF group) and 16 ND CKD patients (8 in each group) completed the study.  Figure 1 presents the CONSORT flow diagram of the study for HD patients, and Figure 2 presents the CONSORT flow diagram of the study for ND patients.

The primary etiology of CKD was hypertension (63.3% of total participants), followed by diabetic nephropathy (9.0%), lithiasis (6.0%), and pyelonephritis (3.0%), and 18.7% of the total participants presented an undetermined etiology. Nine patients with type 2 diabetes and 30 patients with hypertension were similarly distributed between groups. The general characteristics of the participants at baseline are shown in Table 1. As expected, patients on dialysis were younger, and ND CKD patients presented higher BMI than HD patients.  Table 2 shows the biochemical parameters, plasma levels of MDA and inflammatory cytokines (IL-6 and TNF-*α*), and mRNA expression of transcription factors NRF2 and NF-*κ*B at baseline. There were no significant differences between the placebo and TRF intragroup.  Table 3 shows the dietary food intake of the patients studied. Also, as expected, ND CKD patients presented low protein intake. Patients maintained their diets throughout the study.

**Figure 3:**
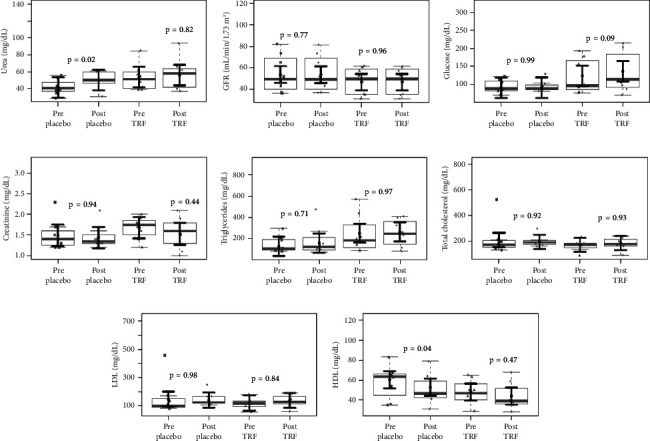
Biochemical and lipid profile parameters in nondialysis patients before and after the intervention. Urea plasma levels were increased in the placebo group (a). There is no evidence of differences in glomerular filtration rate (b), glucose (c), and creatine (d) plasma levels after 3 months of intervention in both groups. Regarding the lipid profile, no differences were observed in triglycerides (e), total cholesterol (f), and LDL (g) plasma levels after 3 months of intervention in both groups. HDL plasma levels were reduced in the placebo group (h). In gray, the sample distributions of data are represented in box plots and strip plots. In black, the center circle represents the mean expected marginal effect for each group estimated from linear mixed-effects models, where patients were considered a random effect. The fixed effects in the models were the intervention group, the time, their interaction, and the confounding variables (e.g., age, sex, and BMI) at the baseline. Black horizontal bars represent the 95% confidence intervals of the expected mean marginal effects by groups. values were corrected for the number of contrasts/two-by-two comparisons by the Tukey honest significant difference (HSD) method.

**Figure 4:**
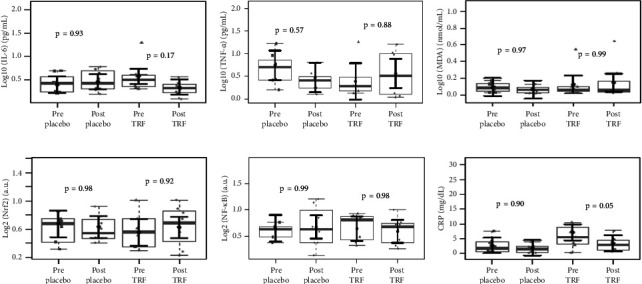
Inflammatory markers, MDA, and transcription factors in nondialysis patients before and after the intervention. There is no evidence of significant alterations in IL-6 (a), TNF-*α* (b), and MDA (c) plasma levels. We also did not find a difference in the log- (bases 2) in mRNA expression levels between groups after 3 months of intervention for NRF2 (d) and NF-kB mRNA expression (e). We found a significant reduction in CRP serum levels (f) after TRF supplementation. In gray, the sample distributions of data are represented in box plots and strip plots. In gray, the sample distributions of data are represented in box plots and strip plots. In black, the center circle represents the mean expected marginal effect for each group estimated from linear mixed-effects models, where patients were considered a random effect. The fixed effects in the models were the intervention group, the time, their interaction, and the confounding variables (e.g., age, sex, and BMI) at the baseline. Black horizontal bars represent the 95% confidence intervals of the expected mean marginal effects by the groups. values were corrected for the number of contrasts/two-by-two comparisons by the Tukey honest significant difference (HSD) method.

**Figure 5:**
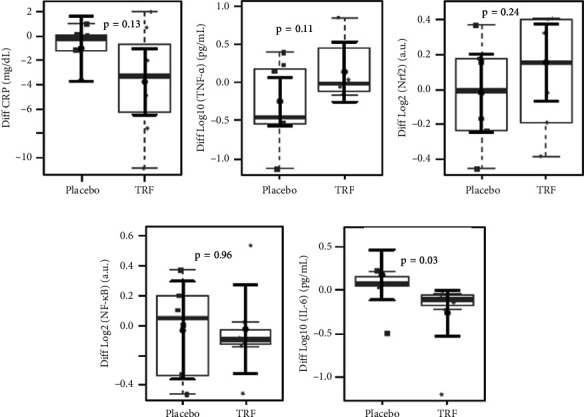
Comparison of inflammation markers in non–dialysis patients between groups after the intervention. We did not find changes in CRP (a) and TNF-*α* (b) plasma levels. Also, no changes were found in NRF2 (c) mRNA expression and NF-kB (d); however, we found a significant reduction in IL-6 plasma levels (e) in the TRF group. In gray, the sample distributions of data are represented in box plots and strip plots. Squares identify placebo, and circles identify TRF differences in plasma levels between 3 months of treatment and baseline. In black, the center circle represents the mean expected marginal effect for each group estimated from linear fixed-effects models. The fixed effects in the models were the intervention group, and the confounding effects were age, sex, and BMI at baseline. Black horizontal bars represent the 95% confidence intervals of the expected mean marginal effects by the group.

Concerning the effect of supplementation of TRF on biochemical parameters in CKD-ND patients, Figure 3 shows a significant increase in the urea plasma levels and a significant reduction in HDL plasma levels in the placebo group, which did not occur in the TRF group. There were no changes in MDA and mRNA expression of transcription factors after the intervention. Plasma levels of IL-6 and TNF-*α* also were not changed; however, CRP serum levels were reduced after TRF supplementation (Figure 4). When comparing inflammation markers in nondialysis patients between groups after the intervention, we observed a reduction in the fold change in IL-6 plasma levels after 3 months of intervention in the TRF group (Figure 5). In addition, there were no changes in routine biochemical parameters after 3 months of intervention (data not shown).

**Figure 6:**
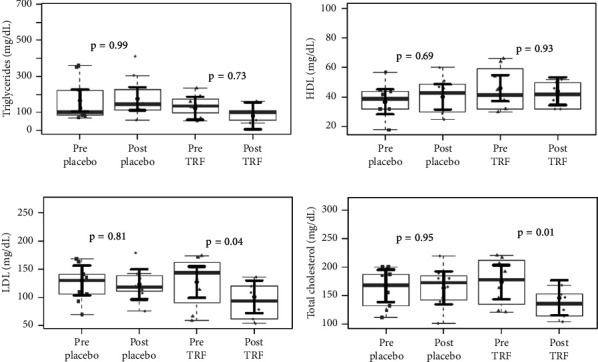
Lipid profile in hemodialysis patients before and after the intervention. There is no evidence of differences in plasma levels of triglycerides (a) and HDL (b) after 3 months of intervention in both groups. Total cholesterol (c) and LDL (d) plasma levels were reduced in the TRF group. In gray, the sample distributions of data are represented in box plots and strip plots. In black, the center circle represents the mean expected marginal effect for each group estimated from linear mixed-effects models, where patients were considered a random effect. The fixed effects in the models were the intervention group, the time, their interaction, and the confounding variables (e.g., age, sex, and BMI) at baseline. Black horizontal bars represent the 95% confidence intervals of the expected mean marginal effects by the groups. *p* values were corrected for the number of contrasts/two-by-two comparisons by the Tukey honest significant difference (HSD) method.

**Figure 7:**
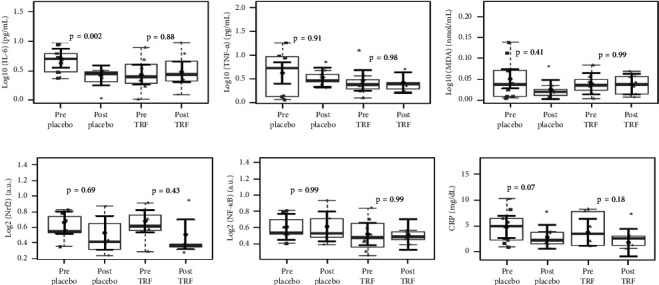
Inflammatory markers, MDA, and transcription factors in hemodialysis patients before and after the intervention. We found a reduction in IL-6 (a) in the placebo group, and we did not find changes in TNF-*α* (b) and MDA (c) plasma levels. We also did not find a difference in the log- (bases 2) in mRNA expression levels between groups after 3 months of intervention for NRF2 (d), NF-kB mRNA expression (e), and CRP plasma levels (f) after TRF supplementation. In gray, the sample distributions of data are represented in box plots and strip plots. In black, the center circle represents the mean expected marginal effect for each group estimated from linear mixed-effects models, where patients were considered a random effect. The fixed effects in the models were the intervention group, the time, their interaction, and the confounding variables (e.g., age, sex, and BMI) at baseline. Black horizontal bars represent the 95% confidence intervals of the expected mean marginal effects by the groups. *p* values were corrected for the number of contrasts/two-by-two comparisons by the Tukey honest significant difference (HSD) method.

**Figure 8:**
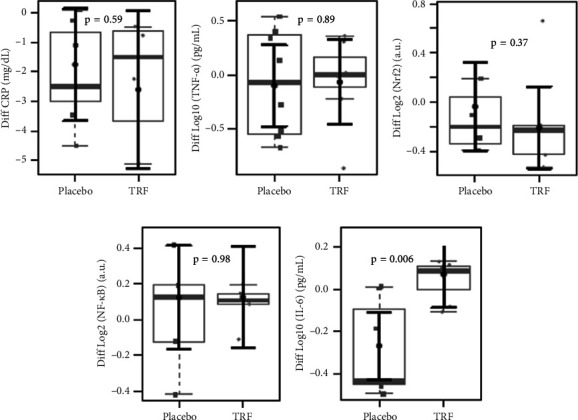
Comparison of inflammation markers in hemodialysis patients between groups after the intervention. We did not find changes in CRP (a) and TNF-*α* (b) plasma levels. Also, no differences were found in NRF2 (c) mRNA expression and NF-kB (d); however, we observed a significant increase in IL-6 plasma levels (e) in the TRF group. In gray, the sample distributions of data are represented in box plots and strip plots. Squares identify placebo, and circles identify TRF differences in plasma levels between 3 months of treatment and baseline. In black, the center circle represents the mean expected marginal effect for each group estimated from linear fixed-effects models. The fixed effects in the models were the intervention group, and the confounding effects were age, sex, and BMI at baseline. Black horizontal bars represent the 95% confidence intervals of the expected mean marginal effects by the group.

Concerning the lipid profile of HD patients, as shown in Figure 6, total cholesterol and LDL plasma levels were significantly reduced after TRF supplementation.  Figure 7 shows the markers of inflammation and oxidative stress in HD patients. In the placebo group, there was a statistically significant decrease in the plasma concentration of IL-6, which did not occur in the TRF group. This difference continues when comparing HD patients' placebo and intervention groups, as seen in Figure 8.

Moreover, the results are further contextualized by the effect sizes and 95% CIs. Among the analyzed outcomes, together with the statistical significance, even a small effect size (Cohen's *d* = 0.29; 95% CI: −0.66–1.25) of the LDL level reduction in HD patients among the examined outcomes (Cohen's *d* = −0.39; 95% CI: −1.35 to 0.58) points to a possible advantage of TRF. A moderate decrease in CRP levels (Cohen's *d* = −0.39; 95% CI: −1.35 to 0.58) was noted in ND patients, suggesting a potential anti-inflammatory impact. There was no effect on MDA levels (Cohen's *d* = 0.00; 95% CI: −0.95 to 0.95), but there was a substantial effect size that showed a trend toward a decrease in IL-6 (Cohen's *d* = −0.95; 95% CI: −1.95 to 0.05) and TNF-*α* (Cohen's *d* = −0.53; 95% CI: −1.50 to 0.44), whereas NRF2 and NF-*κ*B showed modest or negligible effect sizes (Cohen's *d* = −0.21; 95% CI: −1.17 to 0.74 and Cohen's *d* = −0.12; 95% CI: −1.07 to 0.84, respectively). Despite the large CIs, the observed patterns point to a positive impact of TRF supplementation in CKD, especially concerning inflammation and lipid profiles.

## 5. Discussion

Vitamin E is a powerful antioxidant that preserves cellular function by shielding the polyunsaturated fatty acids in the cell membrane from oxidation [24]. Tocotrienols, the vitamin E isomers with an unsaturated side chain, may exert a superior antioxidant effect compared to tocopherols because they effectively neutralize the peroxyl radical and potentially exhibit better distribution within the cell phospholipid bilayer [25]. This study investigated whether TRF supplementation could improve the antioxidant and inflammatory status and mRNA expressions of NF-*κ*B and NRF2 in patients with CKD. This study demonstrated that TRF supplementation in CKD patients had differing effects based on treatment status. TRF significantly reduced LDL cholesterol and total plasma cholesterol levels in patients undergoing HD, improving lipid profiles. Among ND CKD patients, CRP plasma levels decreased significantly. However, expressions of transcription factors NRF2 and NF-*κ*B mRNA remained unchanged in both groups.

NF-*κ*B is kept inactive in the cytoplasm by binding to its inhibitor, I*κ*B (inhibitor of kappa B). Upon stimulation (e.g., by pro-inflammatory cytokines, oxidative stress, or microbial products), I*κ*B undergoes phosphorylation by the I*κ*B kinase (IKK) complex. This phosphorylation targets I*κ*B for ubiquitination and subsequent proteasomal degradation, releasing NF-*κ*B. Freed NF-*κ*B translocates to the nucleus, where it binds to *κ*B response elements in the promoters of target genes. This binding activates the genes' transcription in the immune response, inflammation, cell survival, and proliferation [26]. Meanwhile, NRF2 is kept in the cytosol by its inhibitor Kelch-like ECH-associated protein 1 (Keap1), forming the NRF2-Keap1. The disruption of this system enables NRF2 migration to the nucleus, where it binds to musculoaponeurotic fibrosarcoma, sMaf, and Antioxidant Response Elements to delay the transcription of genes involved in the expression of antioxidant enzymes and cellular protection [27]. TRF promotes the translocation of NRF2 to the nucleus and favors the expression of these genes in a dose-dependent manner [28]. Our results support that the effect on intracellular oxidative regulation pathways might require prolonged intervention or higher dosages.

Closely related to transcription factors and their induced immune responses to different triggers within their activation and translocation to the nucleus is cytokine expression [29]. In our study, supplementation did not influence inflammatory or oxidative markers, such as IL-6, TNF-*α*, and MDA, neither for ND nor HD patients. Still, when comparing groups, a decrease in the fold-change of IL-6 plasma levels was observed in the ND CKD patients group receiving TRF, whereas the HD patients group showed an increase within the TRF group. This emphasizes the complexity of immune responses in CKD and suggests that the reaction to TRF supplementation may differ among patients and their treatments.

While no significant changes in oxidative status or major alterations in pro-inflammatory cytokines were observed, the supplementation showed a notable reduction in CRP plasma levels in ND CKD patients. This protein is involved in acute and chronic inflammation associated with the impairment of nitric oxide production and endothelial cell function, which leads to CVD [30]. It can also enhance platelet aggregation, reduce prostacyclin, and mobilize monocytes into atheromatous plaques [31, 32]. Additionally, an increased CRP is a prognostic indicator for CVD and is also linked to reduced kidney function. Hence, our findings here are noteworthy [30, 33].

Intriguingly, a recent systematic review and meta-analysis of randomized clinical trials focusing on the impact of polyphenols and vitamin E in curcumin/turmeric on oxidative stress and pro-inflammatory biomarkers found that vitamin E supplementation reduced serum CRP levels in CKD patients, with the highlights for patients in chronic dialysis [34]. On the other hand, a randomized clinical trial conducted with HD patients did not observe any alterations in CRP after supplementation with vitamin E (600 IU/day) for 10 weeks. [35]. It is worth mentioning that the TRF was investigated instead of the commonly studied *α*-tocopherol in our study.

A review based on the GRADE approach found that the strength of evidence supporting diverse nutritional supplements to improve kidney function or CKD prognosis was low to very low [36]. Corroborating this, in our study, no improvement was observed in the kidney function parameters with TRF supplementation. Even then, it could still benefit CKD patients by mitigating metabolic conditions [11].

The reduction of total cholesterol and LDL serum levels in HD patients after TRF supplementation underscores the potential of tocotrienols as a vital agent in mitigating dyslipidemia in advanced CKD, which may be attributed to its capacity to inhibit 3-hydroxy-3-methyl-glutaryl-coenzymeA, a pivotal enzyme in cholesterol biosynthesis involved in the conversion of *β *-methyl butyryl-CoA to mevalonate and the later production of cholesterol [37]. Similarly, Daud et al. conducted a trial with HD patients who received TRF capsules containing 180 mg tocotrienols and 40 mg tocopherols to be consumed twice and observed positive effects in participants' lipid profiles, with improvements in triglycerides and HDL after 12 and 16 weeks. Also, a lower cholesteryl-ester transfer protein activity and a higher apolipoprotein A1 concentration were noticed [18]. Tocotrienol supplementation is also associated with LDL reduction by inducing LDL receptor expression [38].

With this study, we confirmed the well-documented clinical effects of supplementation on the lipid profile of HD patients. In this regard, it is essential to note that tocotrienols, liposoluble substances, are transported through chylomicrons. They may reach the liver within 2 h, transported to other organs, and stored in adipose tissue [16, 39, 40]. Therefore, despite the absence of serum dosage, we can infer from previous studies that supplementation was a key factor in this result. Additionally, research has indicated that tocotrienols are more bioavailable when consumed in a fed state [40]. We instructed participants to take capsules after meals, potentially enhancing their absorption.

Nevertheless, despite the possible effect in improving dyslipidemia, TRF did not induce alterations in the lipid profile of ND patients in our study. Patients undergoing diverse treatments for different stages of CKD may vary in metabolic conditions, with more severe impairments observed in HD patients, which may explain the contrasting response among the groups studied here [41]. Indeed, besides contributing to the typical inflammation, oxidative stress, and dysbiosis in CKD, this treatment alters blood hemodynamics [42, 43]. Moreover, it has been suggested that vitamin E can vary its effects under diverse circumstances [16].

We observed a variable response for patients in different conditions, emphasizing the need for a deeper understanding of TRF's unique influence on the study populations. In this regard, some points must be considered. For instance, TRF may have a limited impact on the progression of the disease. Also, despite the common comorbidities such as diabetes and hypertension being similarly distributed among the groups, they may differently influence the absorption and response to supplementation depending on the patient's treatment. Furthermore, we must recognize the interconnection between anti-inflammatory or antioxidant systems, in which vitamin E homologs may act but not be enough on themselves [14].

This study focused on intermediate outcomes, including dyslipidemia and oxidative stress, and inflammation markers, such as TNF-*α*, IL-6, and CRP, which predict cardiovascular health risk [30, 33]. This offers valuable insight into the positive effects of TRF supplementation on these outcomes. Additionally, the study populations did not encounter major adverse cardiovascular events or significant adverse kidney events during the study period. However, the impact of supplementation on these outcomes still needs further examination enlightening.

Moreover, our study has significant limitations, such as the lack of analysis for circulating tocotrienol and tocopherol, which would allow for a more complete understanding of their mechanisms. Additionally, the small sample size and short research duration resulted in wide CIs for various outcomes and limited the results' generalizability. Also, the exploratory nature of the analysis calls for a cautious interpretation of the findings.

Nevertheless, they offer valuable insights into tocotrienol supplementation and its distinct effects in diverse contexts. Moreover, it is worth mentioning that this study was conducted to recruit as many patients as possible. However, it was impacted by the capsules' limited expiration date and the COVID-19 pandemic.

In conclusion, our results support the supplementation of 300 mg of TRF per day for 3 months to improve the lipid profile of HD and the inflammatory status of ND CKD patients as a nutritional strategy to mitigate their cardiovascular risk. However, this finding still needs to be confirmed by larger-scale trials.

## Figures and Tables

**Table 1:** Baseline characteristics of ND and HD patients in placebo and TRF groups.

Parameters	Overall	ND CKD patients		*p* value	HD patients		*p* value
		Placebo group	TRF group		Placebo group	TRF group	
Sex (female/male)	13 (39.4%)/20 (60.6%)	3 (37.5%)/5 (62.5%)	4 (50%)/4 (50%)	1.00	3 (33.3%)/6 (66.7%)	3 (37.5%)/5 (62.5%)	1.00
Age (years)	59 (18)	63.5 (6)	64.5 (7.5)	0.75	53 (16)	47 (16)	0.53
BMI (m/kg^2^)	25 (6.8)	29.8 (6.1)	27.7 (7.68)	0.95	23.4 (2.1)	24.25 (4.57)	0.88

*Note:* Data are presented as either absolute (relative) frequencies or median (IQR). *p* values were estimated by either chi-squared or nonparametric Mann–Whitney *U* tests.

Abbreviations: ND, nondialysis; HD, hemodialysis; BMI, body mass index.

**Table 2:** Baseline biochemical parameters, inflammatory markers, and mRNA expression of transcription factors of ND and HD patients in the placebo and the TRF groups.

Parameters	Overall	ND CKD patients			HD patients		
		Placebo group	TRF group	*p* value	Placebo group	TRF group	*p* value
Urea (mg/dL)	66 (59)	40.5 (7.5)	51.5 (17)	0.11	102 (16)	103.5 (38)	0.53
Creatinine (mg/dL)	3 (7.6)	1.4 (0.3)	1.75 (0.3)	0.17	9.6 (1.4)	8.8 (2.8)	0.59
Glucose (mg/dL)	86 (32)	88.5 (25)	96 (73.5)	0.40	82 (29)	82.5 (42.5)	0.81
GFR (mL/min)	23.7 (37.5)	49.6 (25.28)	49.8 (22.8)	0.38	8.7 (2.9)	10.1 (2.53)	0.48
TG (mg/dL)	135 (108)	108.5 (90.8)	182.5 (143)	0.32	102 (138)	136.5 (52.5)	0.96
TC (mg/dL)	169 (54)	168.5 (45)	169.5 (24.8)	0.79	168 (55)	177.5 (68.3)	0.53
HDL (mg/dL)	44 (27)	63.5 (16)	47 (13.3)	0.18	39 (12)	41.5 (24.5)	0.56
LDL (mg/dL)	119 (61)	100 (51.3)	117.5 (35.8)	0.87	130 (35)	143.5 (56)	0.66
Albumin (g/dL)	3.35 (1.1)	3.2 (0.3)	3.2 (0.3)	0.62	4.3 (0.3)	4.1 (1.4)	0.18
PTH (mg/mL)	131 (310)	113 (37)	73 (32)	0.05	508 (972)	396 (517)	0.42
Hb (g/dL)	11.9 (2.6)	12.8 (1.9)	13.4 (2.7)	0.93	10.3 (2.6)	11.8 (2.1)	0.22
Fe (mg/dL)	78.5 (37)	90 (12.5)	60 (47)	0.62	78 (65)	71 (30.5)	0.71
Ferritin (mg/mL)	365 (432)	213 (72)	283 (175)	0.10	667 (443)	485 (416)	0.76
Calcium (mg/dL)	9.3 (1.3)	9.2 (1.03)	9.75 (0.1)	0.26	8.3 (1.03)	8.6 (1.3)	0.56
Potassium (mg/dL)	5.0 (1.0)	4.4 (0.4)	4.6 (0.6)	0.40	5.2 (0.7)	5.8 (0.8)	0.04
Phosphorus (mg/dL)	4 (1.4)	3.7 (0.5)	3.95 (0.3)	0.24	5.3 (1.6)	4.7 (1.1)	0.32
CRP (mg/L)	3.6 (5.6)	1.8 (3.3)	5.5 (5.8)	0.28	4.9 (4.2)	3.4 (5.6)	0.68
MDA (nmol/mL)	1.1 (1.6)	2.1 (2.2)	1.5 (1.4)	0.87	0.9 (1.6)	0.9 (0.5)	0.92
TNF-*α* (pg/mL)	16.4 (48.9)	40.4 (48.9)	9.2 (15.2)	0.34	43.7 (76.0)	14.3 (7.6)	0.56
IL-6 (pg/L)	20.4 (25.7)	17.0 (20.7)	21.8 (15.5)	0.39	39.9 (31.7)	15 (18.0)	0.06
NRF2 (a.u)	1.1 (0.5)	1.2 (0.6)	1.0 (0.7)	0.74	0.9 (0.4)	1.1 (0.4)	0.44
NF-kB (a.u)	1.0 (0.7)	1.1 (0.4)	1.5 (0.9)	0.53	0.9 (0.4)	0.8 (0.5)	0.33

*Note:* Data are presented as median (IQR). Nonparametric Mann estimated *p* values–Whitney *U* tests.

Abbreviations: a.u., arbitrary unit; CRP, C-reactive protein; Fe, iron; GFR, glomerular filtration rate; HB, hemoglobin; HD, hemodialysis; HDL, high-density lipoprotein; IL-6, interleukin-6; LDL, low-density lipoprotein; MDA, malondialdehyde; ND, nondialysis; NF-kB, nuclear factor kappa B; NRF2, factor 2-related erythroid nuclear factor 2; PTH, parathyroid hormone; TC, total cholesterol; TG, triglycerides: TNF-*α*, tumor necrosis factor-*α*.

**Table 3:** Food intake of HD and ND patients in the placebo and the TRF groups.

Parameters	Overall	ND CKD patients			HD patients		
		Placebo group	TRF group	*p* value	Placebo group	TRF group	*p* Value
Energy (kcal/d)	1550.6 (443.7)	1835.1 (347.9)	1541.5 (296.0)	0.28	1540 (366.5)	1548.9 (535.1)	0.66
Energy (kcal/kg/d)	21.9 (5.2)	24.0 (7.2)	22.4 (5)	0.23	24 (7.1)	21.4 (3.1)	0.28
Protein (g/d)	63.5 (35.1)	54.7 (11.1)	46.9 (13.4)	0.69	74 (64.8)	82.2 (9.8)	1.00
Protein (g/kg/d)	0.8 (2.7)	0.7 (0.2)	0.6 (0.2)	0.57	3.2 (0.9)	4.3 (1.2)	0.12
Lipids (%)	25.2 (5.2)	27.1 (9.9)	27.8 (2.4)	0.15	24.8 (3.2)	24.1 (3.8)	0.73
Carbohydrates (%)	57.5 (13.6)	58 (19.8)	60.2 (18.8)	0.61	56.4 (10.5)	56.1 (5.43)	0.60
Phosphorus (mg/d)	962.3 (506.0)	1131.1 (201.6)	672.7 (215.9)	0.02	878.3 (413.7)	1276.6 (447.4)	0.13
Potassium (mg/d)	2515 (1409)	3678 (901)	3627 (309)	0.95	2333 (642)	2163 (717)	0.80
Vitamin *E* (mg/d)	6.7 (2.2)	6.9 (0.3)	7.3 (1.5)	0.72	6.3 (2.3)	5.0 (1.15)	0.23

*Note:* Data are presented as median (IQR). Nonparametric Mann estimated *p* values–Whitney *U* tests.

## Data Availability

The data supporting the findings of this study are available upon reasonable request from the corresponding author.
